# Mycetoma: The Spectrum of Clinical Presentation

**DOI:** 10.3390/tropicalmed3030097

**Published:** 2018-09-04

**Authors:** Ahmed Hassan Fahal, Suliman Hussein Suliman, Roderick Hay

**Affiliations:** 1 The Mycetoma Research Centre, University of Khartoum, Khartoum 11111, Sudan; 2 Department of Surgery, Faculty of Medicine, University of Khartoum, Khartoum 11111, Sudan; sul.hus.sul@gmail.com; 3 The International Foundation for Dermatology, London W1P 5HQ, UK; roderick.hay@ifd.org

**Keywords:** mycetoma, clinical presentation, review

## Abstract

Mycetoma is a chronic infection, newly designated by the World Health Organization (WHO) as a neglected tropical disease, which is endemic in tropical and subtropical regions. It follows implantation of infectious organisms, either fungi (eumycetomas) or filamentous bacteria (actinomycetomas) into subcutaneous tissue, from where infection spreads to involve skin, bone and subcutaneous sites, leading to both health related and socioeconomic problems. In common with other NTDs, mycetoma is most often seen in rural areas amongst the poorest of people who have less access to health care. The organisms form small microcolonies that are discharged onto the skin surface via sinus tracts, or that can burrow into other adjacent tissues including bone. This paper describes the clinical features of mycetoma, as early recognition is a key to early diagnosis and the institution of appropriate treatment including surgery. Because these lesions are mostly painless and the majority of infected individuals present late and with advanced disease, simplifying early recognition is an important public health goal.

## 1. Introduction

Mycetoma is a unique and troubling neglected tropical disease, endemic in many tropical and subtropical areas. In addition, it is reported from different parts of the world [1,2]. It is a chronic granulomatous subcutaneous inflammatory disease that has a profound and negative impact on numerous medical, health-related and socioeconomic aspects of the lives of patients and communities in the endemic regions [3,4]. The causative organisms of mycetoma are of bacterial or fungal origin and hence these are classified as actinomycetomas and eumycetomas respectively [5,6]. Traumatic implantation of these causative organisms, which are soil and environmental inhabitants, into the subcutaneous tissue via minor trauma or injury is believed to be the route of entry in the development of mycetoma [7,8]. During the evolution of the infection, the causative organisms grow into modified localised microcolonies known as grains that are surrounded by inflammatory cells and give rise to inflammatory sinus tracts. Mycetoma is basically a localised disease; the inflammatory process usually involves the local subcutaneous tissue but it then spreads along the different tissue planes to invade the skin, deep tissues and structures and eventually the bones [9,10]. If not treated appropriately, it can lead to enormous tissue damage, disfigurement and disability that hinder the patients’ normal daily activities [11,12]. Mycetoma patients are unique. They are of low socioeconomic status and health educational level, reside in remote, poor rural areas with meagre medical and health resources. Furthermore, their lesions are usually painless and hence the majority of them present late and with advanced pathology [13,14].

## 2. Age, Gender and Occupation in Mycetoma

No age is exempted from infection but the majority of patients are young adults in the age group of 15–30 years [15,16]. In most of the reported series, children account for 30% of the cases [17]. This has massive implications for the patients, families and communities in the endemic areas as these are the working and wage-earning members of each society and many of these drop out of education due to illness, which increases the pressure on family and community income and resources in households that are already impoverished [18,19].

Male predominance is a constant finding in mycetoma with a gender ratio of 3.7:1 [20,21]. This is commonly attributed to the greater risk of exposure to microorganisms in the soil during outdoor work-related activities. However, in mycetoma-endemic areas, females are also involved in outdoor work activities and thus other genetic or immunological factors cannot be ruled out [22,23].

Mycetoma is seen in most communities in farmers, field labourers and in herdsmen who come in contact with the land, although in endemic areas people from other occupations are affected [24,25].

The patients’ photographs were all obtained following due informed consent procedure.

## 3. The Clinical Presentation

**Figure 1:**
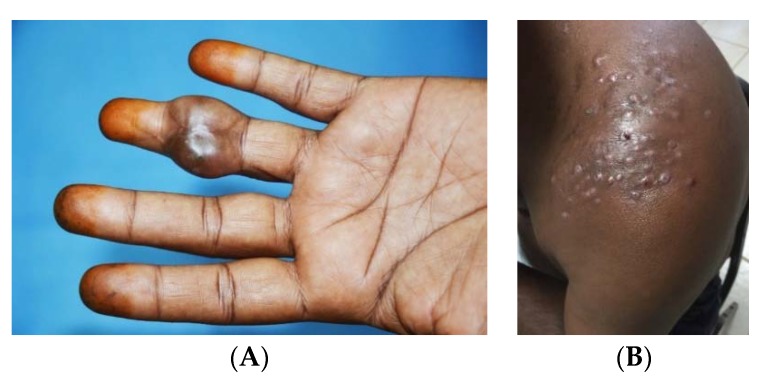
(**A**) A small mycetoma swelling engulfing the middle part of the left ring finger with a scar of previous surgical excision. (**B**) Multiple small nodules of *Nocardia* actinomycetoma at an earlier stage.

**Figure 2:**
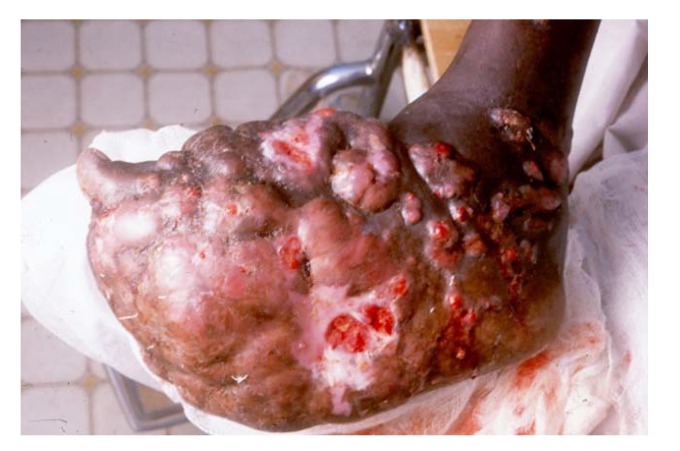
Massive actinomycetoma foot with multiple sinuses and discharge.

**Figure 3:**
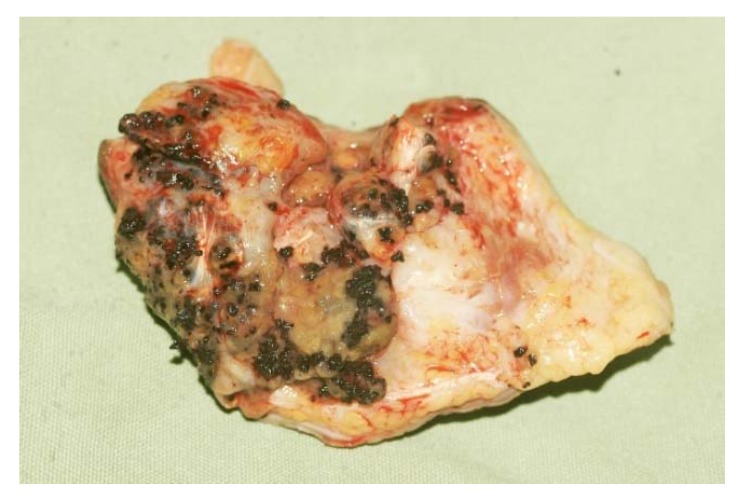
Surgical specimen with multiple black grains.

More than 70 microorganisms are incriminated in causing mycetoma. Nevertheless, the clinical presentation of mycetoma is almost indistinguishable regardless of the causal microorganism [26]. The triad of painless subcutaneous swelling, multiple draining sinuses and discharge that contains grains is characteristics of mycetoma. The swelling is usually firm and rounded but it may be soft, lobulated and, rarely, cystic and it is often mobile (Figure 1) [27]. Multiple secondary nodules then evolve, the nodules may suppurate and drain through multiple sinus tracts, and these sinuses may close transiently after discharge during the active phase of the disease. Prior to discharge, pustules may be visible. Fresh adjacent sinuses may open while some of the old ones may heal completely. The sinuses are connected with each other, with deep abscesses and with the skin surface (Figure 2) [28]. The discharge is usually serous, serosanguinous or purulent. During the active phase of the disease, the sinuses discharge grains, consisting of the microorganisms encapsulated in a cement-like material, melanin and other substances. The grains are believed to protect these microorganisms against host defence mechanisms, antimicrobials and antifungals [29]. The grain colour depends on the causative organism. The grains can be black, yellow, white or red and they are of variable size and consistency. In most mycetomas, they are visible to the naked eye and may be noticed by patients; the exception is actinomycetoma, due to the *Nocardia* species, in which the grains are very small and only visible under the microscope. The black grains are most commonly due to *M. mycetomatis* and related species, the red ones are due to *A. pelletierii*, the yellow to *Streptomyces somaliensis* and the white grains can be due to *A. madurae* [30]. However, black fungi may also produce grains of pale colour. Pus, exudate, the dressing gauze and biopsy material should be examined for the presence of the grains as they may give a clue to the causative organisms although the identity needs to be confirmed as visual inspection alone is not sufficiently accurate (Figure 3).

The onset and progress rate is more rapid with actinomycetoma than with eumycetoma. In eumycetoma, the lesion grows slowly with clearly defined margins and remains encapsulated for a long period, whereas, in actinomycetoma, the lesion is more inflammatory, more destructive and invades the bone at an earlier period; this is more evident in *A. pelletierii* actinomycetoma [31].

**Figure 4:**
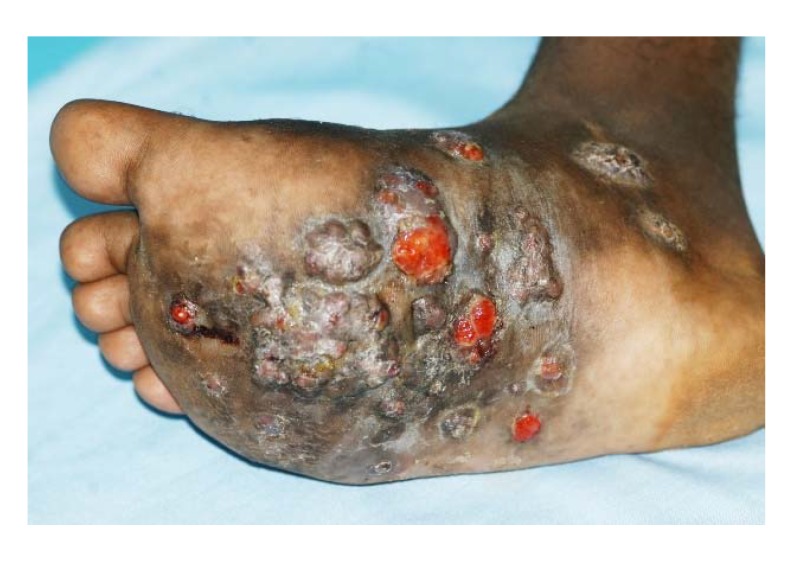
Foot eumycetoma with skin hyperpigmentation, multiple nodules and active sinuses.

**Figure 5:**
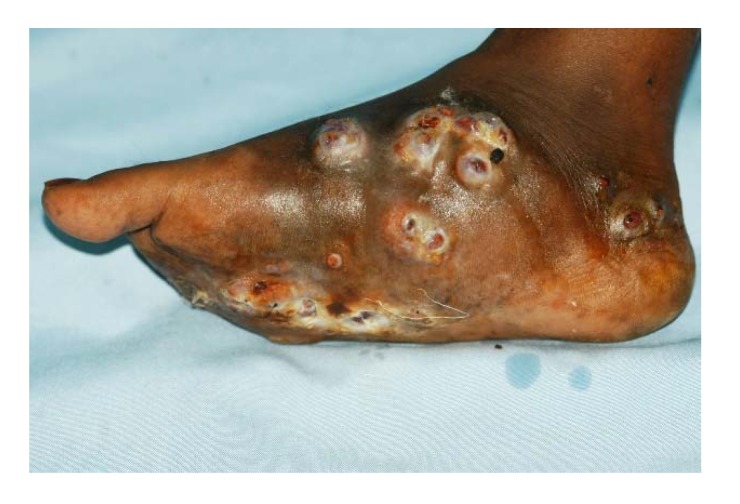
Foot eumycetoma with skin hyperpigmentation, multiple nodules, active sinuses discharging black grains discharge and local hyperhidrosis.

**Figure 6:**
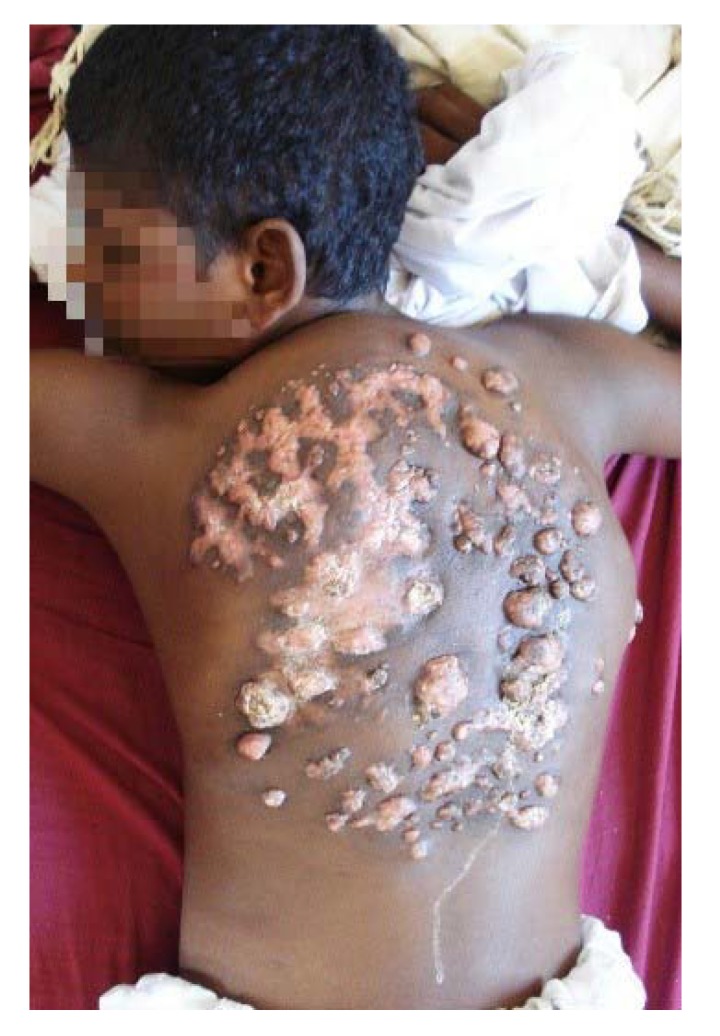
Massive back actinomycetoma with multiple nodules and sinuses.

As the mycetoma granuloma increases in size, the skin over it becomes attached and stretched. The skin may become smooth, shiny and areas of hypo or hyperpigmentation may develop (Figure 4) [32]. In some patients, there may be areas of local hyperhidrosis confined to the mycetoma lesion itself and the skin around it. This is commonly seen with massive lesions in patients with active disease and the cause is unclear but increased local temperature due to brisk arterial circulation may a cause (Figure 5 and Figure 6). In *Nocardia* actinomycetomas the chest wall or back is often affected and here the edges of the lesions are unclear and hard to define.

**Figure 7:**
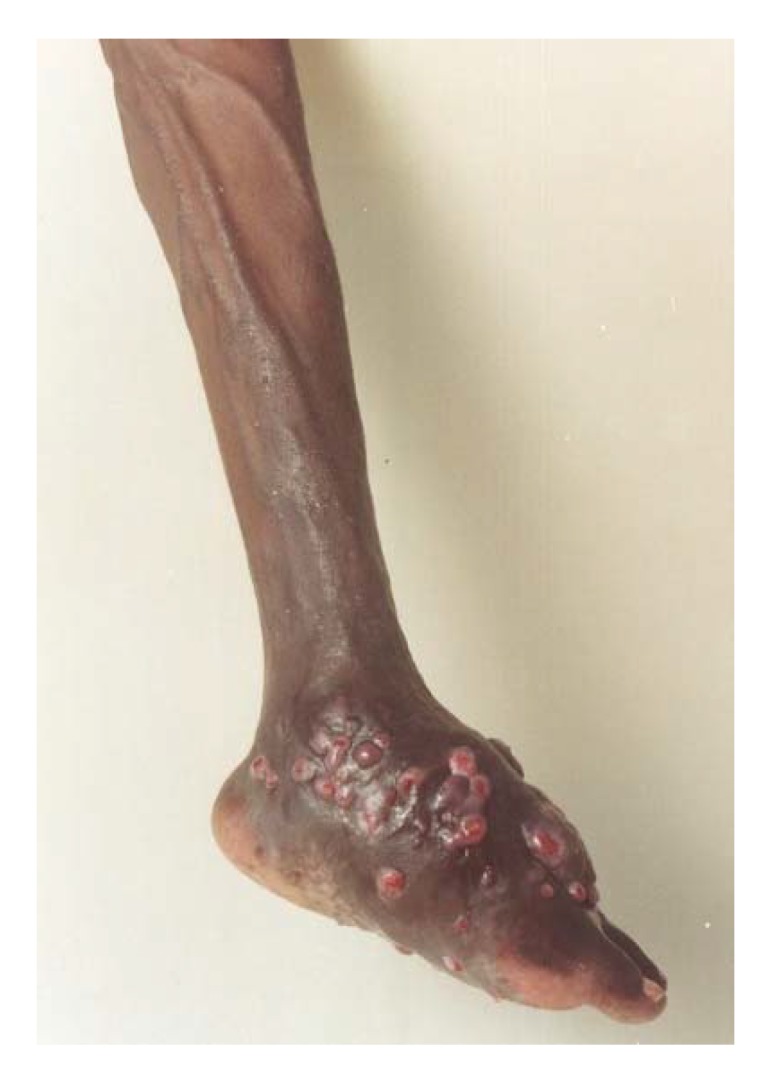
Massive mycetoma foot with dilated tortuous veins at and proximal to the mycetoma site.

In long-standing massive mycetoma lesions, dilated tortuous veins at, and proximal to, the mycetoma site are noted. They are frequently confused with varicose veins. They develop as a secondary phenomenon to accommodate the brisk venous return due to the increased arterial blood flow caused by the chronic inflammatory process at the mycetoma site (Figure 7) [33].

Unusually and for unknown reasons, the tendons and the nerves are spared until very late in the disease process; this may explain the rarity of neurological and trophic changes even in patients with long-standing mycetomas. The absence of trophic changes may be explained by the more than adequate blood supply in the mycetoma-infected area [34].

In the majority of patients, the regional lymph nodes are small and shotty. An enlarged regional lymph node is not uncommon and this may be due to secondary bacterial infection, the genuine lymphatic spread of mycetoma or it may be due to immune complex deposition as part of a local immune response to mycetoma infection [35].

In general, the infection remains localised and constitutional disturbances are rare but when they do occur they are generally due to secondary bacterial infection of the open sinus tracts and generalised immunosuppression. Cachexia and anaemia may be seen in late mycetoma. This is often due to malnutrition, sepsis and mental depression [36]. Co-morbidities in mycetoma are rare and the commonest are diabetes mellitus and hypertension, as in the general population. HIV and malignant transformation are not reported in mycetoma [30].

Mycetoma is usually painless in nature; it has been suggested that the mycetoma produces substances that have an anaesthetic action. At a late stage of the disease, the pain may also become negligible due to nerve damage by the tense fibrous tissue reaction, endarteritis obliterans or poor vascularisation of the nerves [37]. Pain at the mycetoma site is reported in 20% of patients and it is commonly produced by the secondary bacterial infection or transiently when a new closed sinus is about to open onto the skin surface.

**Figure 8:**
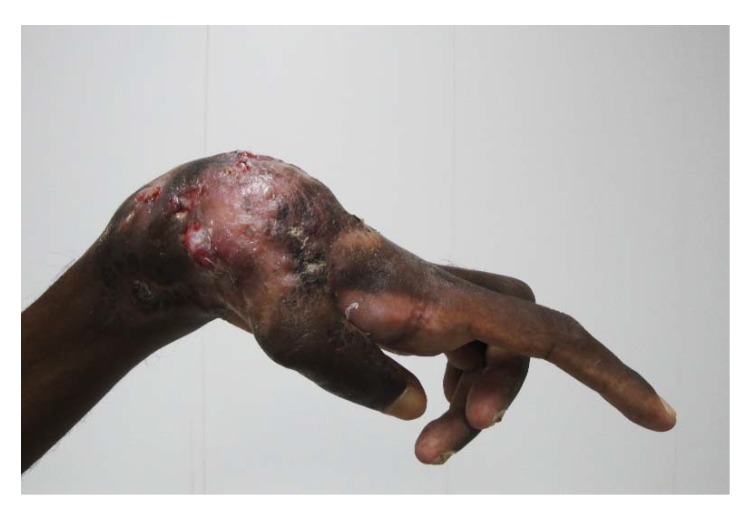
Hand eumycetoma with massive deformity.

Mycetoma can produce many distortions, deformities and disabilities and that is due to structural impairment that includes bone destruction or periostitis, loss of function and disuse atrophy of the affected limb. The current treatment options are limited and suboptimal; many patients undergo repeated massive surgical excisions leading to more tissue destruction and fibrosis and disability (Figure 8) [38].

## 4. The Site of Mycetoma

**Figure 9:**
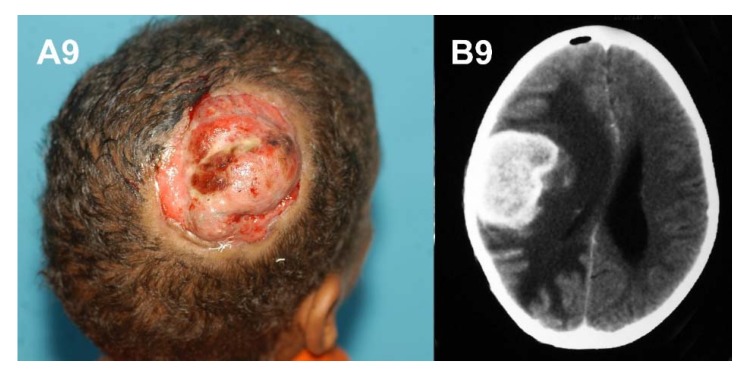
(**A**) massive head eumycetoma. (**B**) CT scan showing intra-cranial extension.

**Figure 10:**
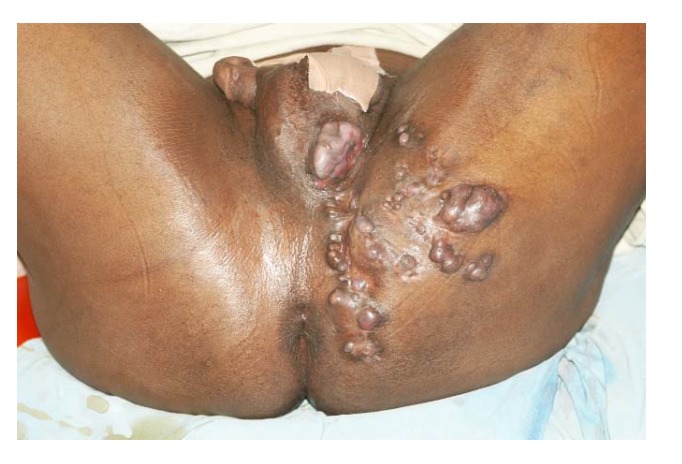
Massive gluteal, perineal and scrotal eumycetoma.

**Figure 11:**
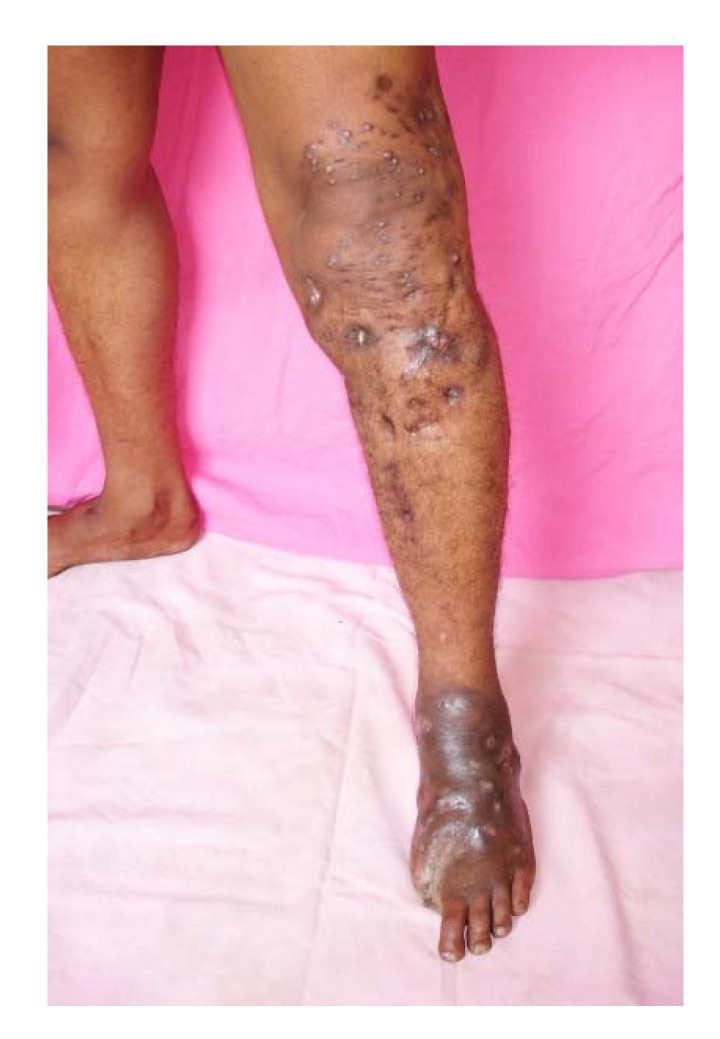
Massive foot actinomycetoma with leg and knee inguinal satellite lesions.

The commonest site for mycetoma is the foot (79.2%); most of the lesions are seen on the dorsal aspect of the forefoot and the left foot is affected slightly more often [39]. The hand ranks as the second commonest site (6.6%), with the right hand is more affected. This may imply a traumatic basis of the infection in this site [40]. In endemic areas, other parts of the body may be involved but less frequently, and these include the knee, arm, leg, head and neck, thigh and the perineum [41,42,43,44,45]. Rarer sites for eumycetomas are the chest and abdominal walls, facial bones, mandible, paranasal sinuses, eyelid, vulva, orbit and scrotum (Figure 9, Figure 10 and Figure 11) [46,47,48,49,50]. *Nocardia* actinomycetomas are often found on the chest or abdominal wall. Bilateral limb mycetomas, although described, are very rare.

## 5. Mycetoma Spread

**Figure 12:**
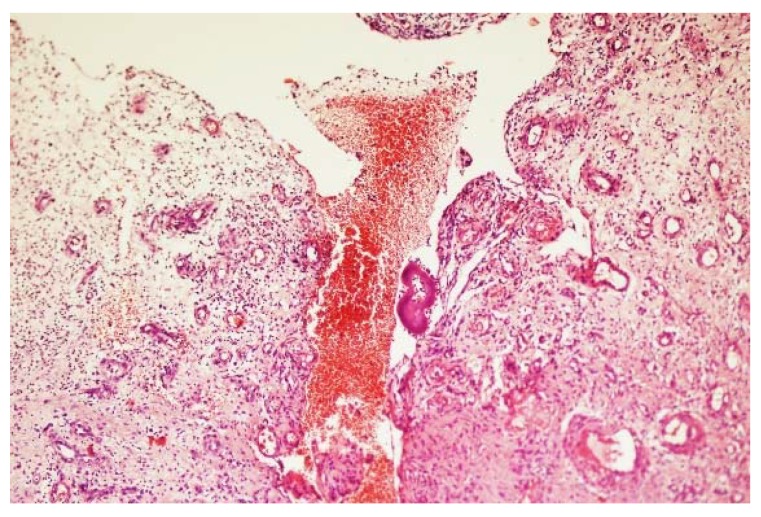
Photomicrograph showing *A. madurae* grains within the lumen of the intact blood vessel (H&E X 200).

Spread along the lymphatics to the regional lymph nodes is reported and this is more frequent with actinomycetoma [12]. At the regional lymph nodes, the disease progresses; a new disease satellite lesion develops and progresses to form a massive mycetoma, and in some cases, this can cause lymphatic obstruction and lymphedema (Figure 11) [51]. Bloodstream spread in mycetoma is a rare event. It has been reported in some cases, where there was spinal cord compression and paraplegia. In these cases, the skin and subcutaneous tissue were normal and the mycetoma was an unexpected finding at surgery upon opening the spinal dura matter [52]. In these cases, the mycetoma grains were actually seen in the lumen of the intact blood vessel (Figure 12).

## 6. Aggressive Mycetoma

Although mycetoma is primarily a localised disease of gradual onset and slow progress, some patients present with massive, aggressive, uncontrolled disease and most of these are fatal. In these patients, the disease progressed wildly and aggressively from the subcutaneous tissue to involve the deep organs such as the urinary bladder, pelvic organs, spinal cord, lung and other structures and resulted in fatal outcome [53,54,55,56].

Two patients with fatal eumycetoma presented with pulmonary secondaries were reported; one from the knee and the second from gluteal eumycetoma. In these patients, *Madurella mycetomatis* had progressed widely without response to the different medical and surgical treatment modalities. Vascular spread, which is a rare phenomenon in mycetoma, may explain the secondary lung lesions encountered in these patients [52,54,55,56].

Several cases of mycetoma of the head and neck region have reported. They proved to be serious medical and health problem, with low cure rate and were associated with grave complications and poor outcome due to intra-cranial spread [57].

**Figure 13:**
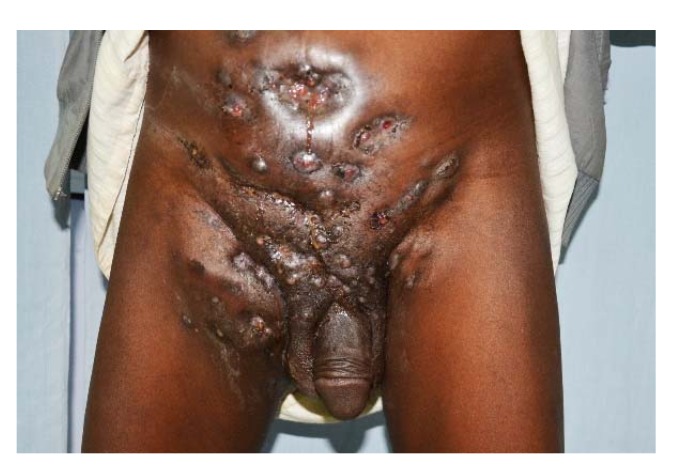
Massive anterior abdominal eumycetoma spreading to both inguinal regions and scrotum.

Pelvic mycetoma spread has also been reported. In these cases, the urinary bladder, rectum, hip bones and other local structures were involved. The disease was complicated by multiple urinary and rectal fistulae, pathological fractures and all patients died from massive sepsis (Figure 13) [52,53,54,55,56].

## 7. The Differential Diagnosis

**Figure 14:**
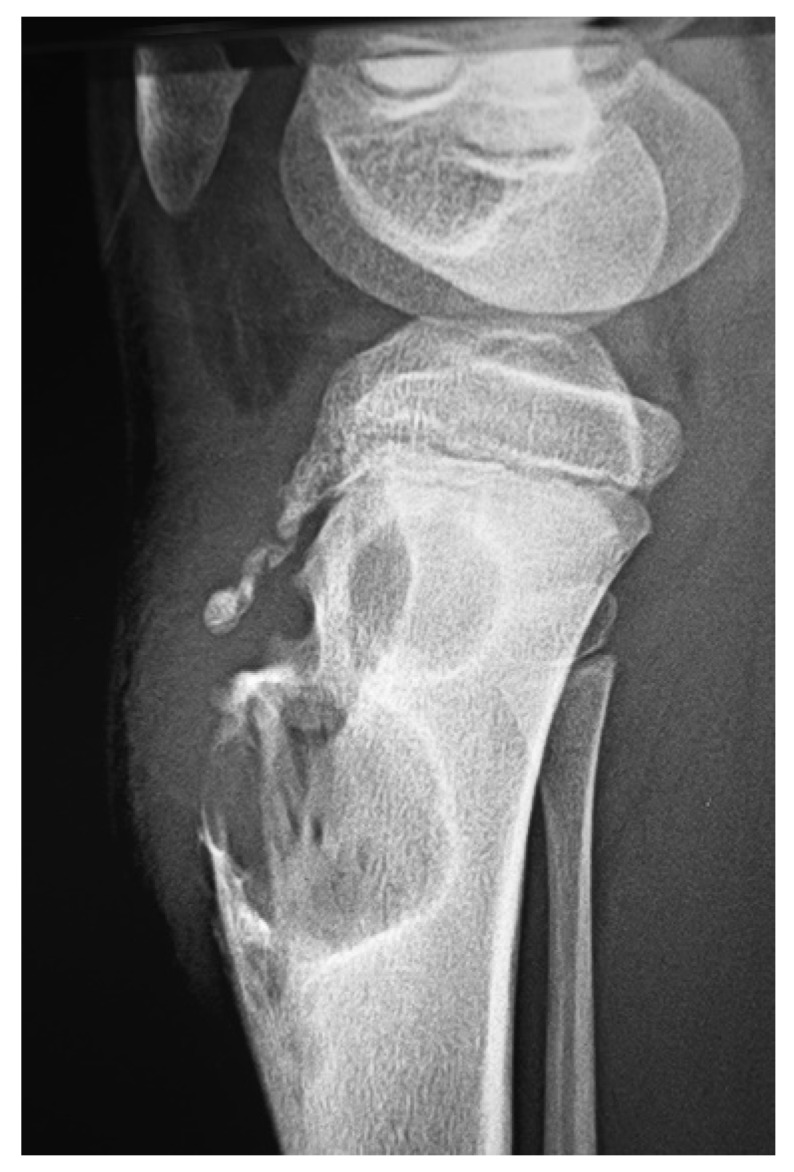
X-ray of thigh and knee eumycetoma showing massive soft tissue shadow, periosteal reaction and multiple bone cavities resembling osteogenic sarcoma.

In endemic areas, any subcutaneous mass is considered as mycetoma unless proved otherwise. The differential diagnosis of mycetoma includes thorn and foreign body granulomas, particularly in mycetoma endemic areas [57]. Also, it includes many soft tissue tumours such as Kaposi’s sarcoma, fibroma, malignant melanoma or fibrolipoma and keloids [1,58]. Many dermatological conditions, such as botryomycosis, sporotrichosis and plantar or acral psoriasis, can mimic mycetoma [59]. In very early mycetomas, before the first appearance of sinuses, the lesion simply resembles a firm subcutaneous nodule, which makes recognition of the earliest signs based on a visual appearance very difficult as the differential encompasses a variety of benign and inflammatory conditions, from dermatofibromas to hypertrophic scars. The radiological features of advanced mycetoma resemble osteogenic sarcoma and bone tuberculosis (Figure 14). Primary osseous mycetoma, without overlying sinuses, is uncommon but must be differentiated from chronic osteomyelitis, osteoclastoma, bone cysts and syphilitic osteitis.

This paper has concentrated on the variations in the clinical presentation of mycetomas. The currently available treatments for mycetoma are suboptimal and unsatisfactory, characterised by a low cure rate and high patient follow-up dropout and recurrence rates [49]. Furthermore, presently there are neither preventive nor control measures or programs for mycetoma. Moreover, mycetoma affects the most underprivileged population in remote areas and hence most patients present late with advanced disease. To overcome this, early case detection, which depends on detecting the visual warning signs of the disease and treatment is the only available means to prevent the numerous and serious complications of mycetoma. The first step in early case detection is meticulous clinical examination of the suspected patients, as other diagnostics tools, including direct microscopy, culture, histopathology and molecular identification, are commonly not available in mycetoma- endemic regions [60].
